# rhPDGF-BB Promotes Proliferation and Osteogenic Differentiation of Bone Marrow Stromal Cells from Streptozotocin-Induced Diabetic Rats through ERK Pathway

**DOI:** 10.1155/2014/637415

**Published:** 2014-01-29

**Authors:** Yanfang Zhao, Songmei Zhang, Deliang Zeng, Lunguo Xia, Ashwini Lamichhane, Xinquan Jiang, Fuqiang Zhang

**Affiliations:** ^1^Department of Prosthodontics, Ninth People's Hospital Affiliated to Shanghai Jiao Tong University School of Medicine, 639 Zhizaoju Road, Shanghai 200011, China; ^2^Department of Stomatology, Shanghai East Hospital Affiliated to Tongji University, Shanghai 200011, China; ^3^Shanghai Stomatological Diseases Center, Shanghai 200001, China; ^4^Department of Oral and Maxillofacial Surgery, Ninth People's Hospital Affiliated to Shanghai Jiao Tong University School of Medicine, Shanghai 200011, China; ^5^Oral Bioengineering Laboratory/Regenerative Medicine Lab, Shanghai Key Laboratory of Stomatology, Shanghai Research Institute of Stomatology, Ninth People's Hospital Affiliated to Shanghai Jiao Tong University School of Medicine, 639 Zhizaoju Road, Shanghai 200011, China

## Abstract

Management of nonunion fracture and massive segmental bone defects in diabetes remains a challenging clinical problem. Bone marrow stromal cells (BMSCs) are crucial for bone remodeling and hold promise for bone regeneration. However, we have showed previously that diabetes can affect the proliferation and osteogenic potential of BMSCs adversely and a strategy to attenuate the impaired functions of BMSCs is required. Platelet-derived growth factor-BB (PDGF-BB) plays an important role in bone formation. However, little information is available about its effect on diabetic BMSCs. In this study, BMSCs were isolated from streptozotocin-induced diabetic rats. After treatment with recombinant human PDGF-BB (rhPDGF-BB), diabetic BMSCs demonstrated enhanced cell proliferation and osteogenic differentiation based on increased expressions of osteogenic genes (Runx2, alkaline phosphatase, and osteocalcin) and Runx2 protein, as well as upregulated alkaline phosphatase activity and mineralization. Furthermore, blocking extracellular signal regulated kinase (ERK) pathway by inhibitor PD98059 repressed the enhanced proliferation and osteogenic differentiation in diabetic BMSCs induced by rhPDGF-BB. Together, these results indicated that rhPDGF-BB stimulates proliferation and osteogenic differentiation partially through ERK pathway in diabetic BMSCs. Therefore, modulation of diabetic BMSCs could augment BMSCs function affected by diabetes and holds significance for future strategies to treat diabetic bone complications.

## 1. Introduction

Type 1 diabetes mellitus is an increasingly prevalent systemic disease in the world. In 2012, the prevalence of diabetes was estimated to be 8.3% worldwide, representing approximately 371 million people living with diabetes [1]. It is well documented that diabetes could contribute to osteoporosis, decreased bone mineral density, increased risk of fracture, and compromised fracture healing rates and bone repair quality [2–6]. The relative risk for hip fracture was as much as 6-7 times higher in patients with diabetes compared with the nondiabetic population [3, 7]. However, to date, fracture nonunion and bone defects in diabetes are still challenging in the clinic.

Bone continuously undergoes a process of self-renewal and repair termed bone remodeling which is involved with bone forming and bone resorbing [8]. Increasing evidence suggests that skeletal abnormalities in type 1 diabetes may partially result from the detrimental effects of diabetes on bone formation [9–11]. Bone marrow stromal cells (BMSCs) are a major source of osteoblasts and are crucial for bone remodeling and repair through direct regeneration such as cell differentiation or maturation, or indirect mechanisms via paracrine effects such as increased vascularization [12]. Besides, BMSCs are easily obtained and expanded in vitro, and they are self-renewing, multipotent stem cells that have the capability of differentiating into osteoblasts, chondrocytes, adipocytes, tenocytes, and myoblasts. As their easy availability and multipotent potential, BMSCs are demonstrated as an attractive candidate for tissue engineering applications, which has been effectively used to enhance bone repair and regeneration [13]. However, we and the other group previously showed that BMSCs derived from streptozotocin-induced diabetic rats exhibited decreased proliferative ability and deficiency in osteogenic differentiation compared with control BMSCs. Also, the above function impairments may be responsible for bone disorders associated with diabetes [14, 15]. Furthermore, these problems could severely affect cell-based therapies for the treatment of diabetic bone diseases. So it is necessary to priming BMSCs to enhance their affected functions.

Platelet-derived growth factor (PDGF) consisting of five types of dimerism (PDGF-AA, PDGF-BB, PDGF-AB, PDGF-CC, and PDGF-DD) is a potent chemoattractant and mitogen that could promote wound and bone healing [16]. Especially, reduced PDGF expression was observed in the diabetic fracture callus, possibly indicating impaired platelet function/aggregation and a corresponding decrease in cell proliferation [17]. Recombinant human PDGF-BB (rhPDGF-BB) is FDA-approved for periodontal regeneration [18]. Numerous studies indicated that rhPDGF-BB could enhance proliferation and osteogenesis of diverse cell types, as well as stimulate bone formation in fracture and bone defects models [19–22]. In particular, local administration of rhPDGF-BB could enhance fracture healing in diabetic rats [23].

Although increased proliferation and osteoblast differentiation in many types of cells induced by rhPDGF-BB have been widely reported previously, little information is available about its effect on diabetic BMSCs. In this study, we investigated the influence and underlying mechanisms of rhPDGF-BB involved in the regulation of proliferation and osteogenesis of BMSCs derived from diabetic rats. This information may shed light on the effect of rhPDGF-BB on bone metabolism and its mechanism in counteracting bone disorders in diabetes.

## 2. Materials and Methods

### 2.1. Recombinant Proteins and Antibodies

rhPDGF-BB was provided by BioMimetic Therapeutics, Inc. (Franklin, TN, USA). The MEK inhibitor PD98059 was purchased from Cell Signaling Technology (Beverly, MA, USA). The monoclonal antibodies used in this study include the following: phosphorylation extracellular signal regulated kinase (p-ERK), total ERK (Cell Signaling Technology, MA, USA), **β**-actin (Sigma, St. Louis, MO, USA), and runt-related transcription factor 2 (Runx2) (bioworld, MN, USA).

### 2.2. Animal Models

All animal experiments were approved by the Animal Research Committee of the Ninth People's Hospital affiliated to Shanghai Jiao Tong University, School of Medicine. Eight 4-week-old male Wistar rats were used in the study. Diabetes was induced via a single intraperitoneal injection of streptozotocin (65 mg/kg) (Sigma, St. Louis, MO, USA) dissolved in 0.01 M citrate buffer (pH = 4.5) as previously described [14]. The diabetic rats were confirmed if their blood glucose concentrations were higher than 16.7 mmol/L tested by a blood glucose meter (Accu-Chek Perferma, Roche Diagnostics, Indianapolis, IN, USA) 1 week after-injection [24]. The blood glucose concentrations and body weights were measured in the same day before injection, 1 week, and 12 weeks after-injection (data not shown). All animals were fed with a regular diet and water ad libitum.

### 2.3. Isolation and Culture of Rat BMSCs

After euthanasia, BMSCs were harvested from the tibia and femur bone marrow of diabetic rats and cultured in low glucose Dulbecco's Modified Eagle Medium (DMEM, low glucose; Gibco, Grand Island, NY, USA) supplemented with 10% fetal bovine serum (Hyclone, Logan, UT, USA), containing 100 U/mL penicillin, 100 U/mL streptomycin, and 2 mM L-glutamine (Sigma) as previously described. After BMSCs were incubated for 24 hours, the medium was changed to discard nonadherent cells. Then the medium was refreshed every 3 days. When confluence reached approximately 80%, cells were detached and passaged. Cells at passage 2 or 3 were used for all experiments. To study the osteogenic differentiation of BMSCs, osteogenic medium containing 10–8 M dexamethasone, 50 **μ**g/mL L-2-ascorbic acid, and 10 mM **β**-glycerophosphate were used.

To examine the effect of rhPDGF-BB on proliferation and osteogenic differentiation of diabetic BMSCs, the cells were divided into 3 groups: control, 10 ng/mL rhPDGF-BB, and 50 ng/mL rhPDGF-BB. For analysis of the role of ERK signaling in the aforementioned effect of rhPDGF-BB, the divided groups were as follows: control, 10 ng/mL rhPDGF-BB, and 10 ng/mL rhPDGF-BB + PD98059.

### 2.4. Cell Proliferation Assay

MTT assay was used to assess the amount of viable cells. Briefly, BMSCs were cultured in 96-well plates at 3 × 10^3^ cells/well initial density, treated with or without rhPDGF-BB or PD98059. After the indicated culture periods, MTT (5 mg/mL) was added into each well and incubated for 4 hours at 37°C. After removing medium, DMSO was used to dissolve formazan and the absorbance value was measured using a microplate reader (Bio-tek, Vermont, USA) at 490 nm.

### 2.5. Real-Time PCR Analysis

At 7 days, total RNA was extracted from each group using Trizol reagent (Invitrogen, Carlsbad, CA, USA) and was transcribed with PrimeScript RT reagent kit (TaKaRa, Kyoto, Japan). The cDNA amplification and detection was performed with the Bio-Rad iQ5 real-time PCR system (Bio-Rad, Hercules, CA, USA) using SYBR Premix Ex Taq Kit (TaKaRa) and specific primers previously used [14]. Then the relative gene expression level was normalized to the internal control (**β**-actin) based on the 2^−ΔΔCt^ method.

### 2.6. Alkaline Phosphatase (ALP) Staining and ALP Activity Assay

Cells were plated in 12-well plates at a density of 2 × 10^4^ cells/well and incubated for 7 or 14 days. For ALP staining, after being fixed for 15 min at 4°C with 10% formalin, BMSCs were treated with a BCIP/NBT solution (Beyotime, Shanghai, China) in the dark, and areas stained purple were regarded as positive. Photomicrographs of each group were captured with a light microscope (Olympus). ALP activities were determined using p-nitrophenyl phosphate (sigma) as described previously [25].

### 2.7. Mineralization Measurement

Cells were plated into a 12-well dish at a density of 2 × 10^4^ cells/well and incubated for 21 days. For alizarin red staining, BMSCs were fixed in 70% ethanol for 1 h and stained with 40 mM alizarin red S solution for 30 min at room temperature. Nonspecific staining was removed by several washes in distilled water. For von Kossa staining, after being fixed in 10% formalin for 15 min, cells were stained with 5% silver nitrate and placed under ultraviolet light for 30 min. Then cells were treated with 5% NaS_2_O_3_ for 2 min and washed with distilled water [26]. Photomicrographs of each group were captured with a light microscope (Olympus).

### 2.8. Western Blot Analysis

For Western blotting, different groups of cells were lysated with a protein extraction regent containing protease inhibitor cocktail, phosphatase inhibitor cocktail and phenylmethanesulfonyl fluoride (PMSF) (Kangchen, Shanghai, China). The protein concentration was measured according to the BCA protein assay kit manufacturer's protocol (Beyotime, Shanghai, China). Equal protein samples were separated on SDS-polyacrylamide gel electrophoresis (PAGE) and then electrotransferred to a polyvinylidene difluoride membrane. The membranes were blocked and primary antibodies (rabbit anti-rat Runx2, 1 : 500; rabbit anti-rat ERK, 1 : 1000; rabbit anti-rat p-ERK, 1 : 1000; mouse anti-rat **β**-actin, 1 : 1000) were incubated. Finally, the blots were visualized with horseradish peroxidase (HRP)-conjugated goat anti-rabbit or anti-mouse IgG (1 : 1000) using the ECL plus reagents (Amersham Pharmacia Biotech, Arlington Heights, USA) by an UVItec ALLIANCE 4.7 gel imaging system. The relative integrated density of each protein band was analyzed by NIH image J 1.34s.

### 2.9. Statistical Analysis

Data were expressed as mean ± standard deviation (SD). All experiments were replicated at least three times. One-way analysis of variance (ANOVA) was performed with post hoc SNK for multiple comparisons (SPSS Inc., Chicago, USA). And *P* < 0.05 was considered to be statistically significant.

## 3. Results

### 3.1. rhPDGF-BB Promoted the Proliferation of Diabetic BMSCs

**Figure 1:**
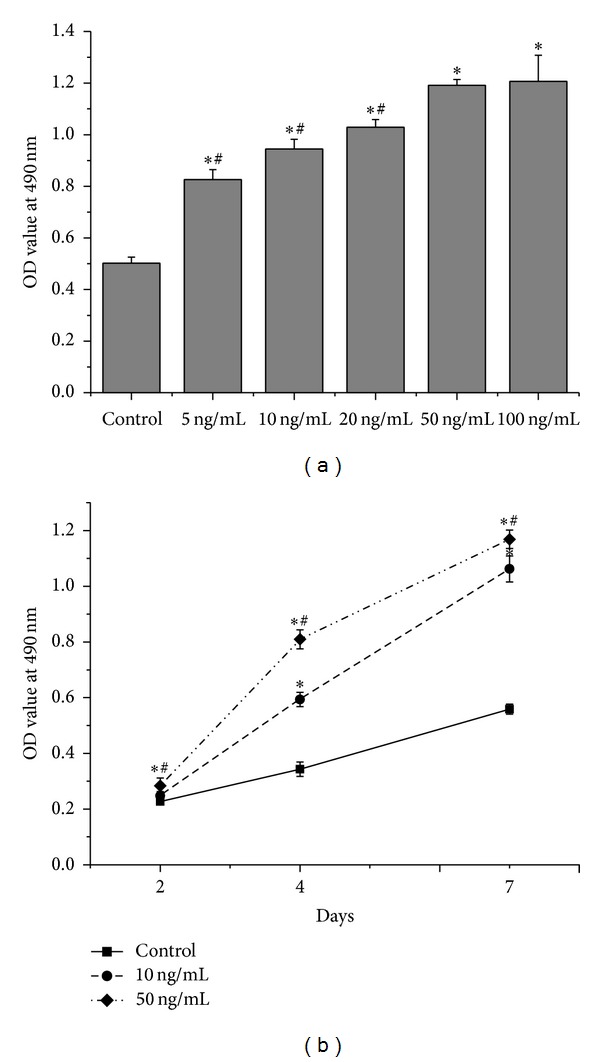
Effects of rhPDGF-BB on proliferation of diabetic BMSCs. (a) MTT assay results of diabetic BMSCs treated with or without rhPDGF-BB (5–100 ng/mL) for 7 days, *P < 0.05, versus control, and ^#^ *P* < 0.05, versus 50 ng/mL. (b) MTT assay results of diabetic BMSCs treated with or without rhPDGF-BB (10 ng/mL or 50 ng/mL) for 2, 4 and 7 days, *P < 0.05, versus control, and ^#^ *P* < 0.05, versus 10 ng/mL.

The MTT assay was performed to investigate whether rhPDGF-BB has positive effect on the proliferation of diabetic BMSCs.  Figure 1(a) showed that cell proliferation significantly increased in groups treated with 5–100 ng/mL rhPDGF-BB compared to negative controls (*P* < 0.05), but there was no significant difference between 50 ng/mL group and 100 ng/mL group in OD values. Moreover, Figure 1(b) revealed that cells cultured in 10 ng/mL and 50 ng/mL rhPDGF-BB groups grew faster than cells cultured in control group on days 2, 4, and 7. The above results indicated that rhPDGF-BB promoted proliferation of diabetic BMSCs in a time- and dose-dependent manner.

### 3.2. rhPDGF-BB Enhanced the Osteogenic Differentiation of Diabetic BMSCs

**Figure 2:**
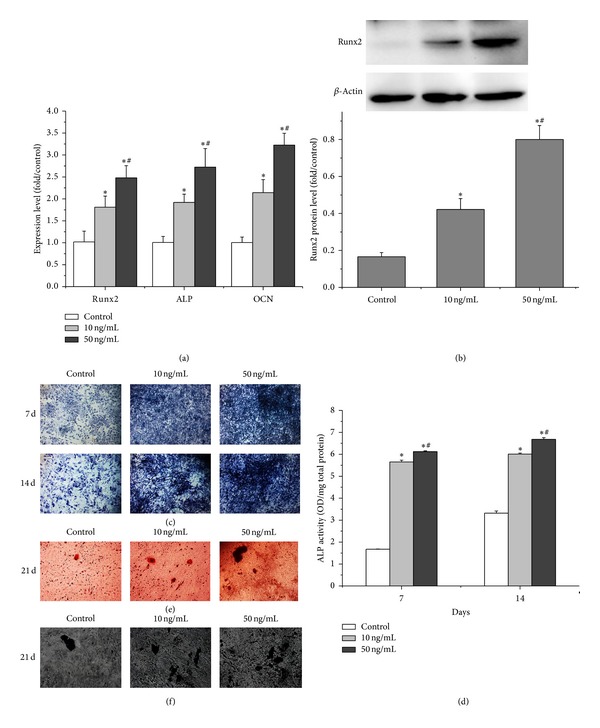
Effects of rhPDGF-BB on osteogenic differentiation of diabetic BMSCs. Cells were treated with or without rhPDGF-BB (10 ng/mL or 50 ng/mL) for the indicated periods. (a) Real-time PCR analysis of ALP, Runx2, and OCN mRNA expression. (b) Western blot analysis for the relative Runx2 protein levels. (c) ALP staining on days 7 and 14 (16x). (d) Quantitative results of ALP assay on days 7 and 14. (e) Alizarin red staining for mineralized nodules on day 21 (50x). (f) von Kossa staining for mineralized nodules on day 21 (50x). *P < 0.05, versus control, and ^#^ *P* < 0.05, versus 10 ng/mL.

Firstly, real-time PCR was performed to evaluate the expression levels of osteogenic genes including Runx2, ALP, and osteocalcin (OCN) on day 7. Compared to the control group, rhPDGF-BB increased the relative expression of Runx2 by 78% and 143%, the ALP by 91% and 171%, and the OCN by 113% and 221%, with respect to 10 ng/mL and 50 ng/mL PDGF-BB group, respectively (Figure 2(a)). Consistent with increased Runx2 mRNA levels, Runx2 protein level was also increased in 10 ng/mL and 50 ng/mL rhPDGF-BB group compared with the control group on day 7, which was detected by western blot analysis (Figure 2(b)). Moreover, there was significant difference between 10 ng/mL group and 50 ng/mL group (Figure 2(b)).

To further evaluate the osteogenic differentiation ability, ALP activity and matrix mineralization ability were analyzed at the defined time points. On days 7 and 14, more intense ALP staining was observed in BMSCs treated with rhPDGF-BB groups than in the control BMSCs, while the most intense ALP staining was observed in the 50 ng/mL group (Figure 2(c)). Similarly, at days 7 and 14, rhPDGF-BB (10 ng/mL and 50 ng/mL rhPDGF-BB groups) significantly increased ALP activity compared with the control group in a dose-dependent manner (Figure 2(d)).

In addition, the Alizarin red staining of mineralized bone nodules on day 21 showed that rhPDGF-BB (10 ng/mL and 50 ng/mL rhPDGF-BB groups) significantly increased matrix mineralization compared with untreated group, while the 50 ng/mL group presented the strongest staining among all groups (Figure 2(e)). Consistent with alizarin red staining results, von Kossa staining also showed the same trend similar to results of Alizarin red staining between the rhPDGF-BB groups and the control group (Figure 2(f)). Taken together, our data revealed that rhPDGF-BB promoted osteogenic differentiation of diabetic BMSCs.

### 3.3. rhPDGF-BB Activated ERK Pathway in Diabetic BMSCs

**Figure 3:**
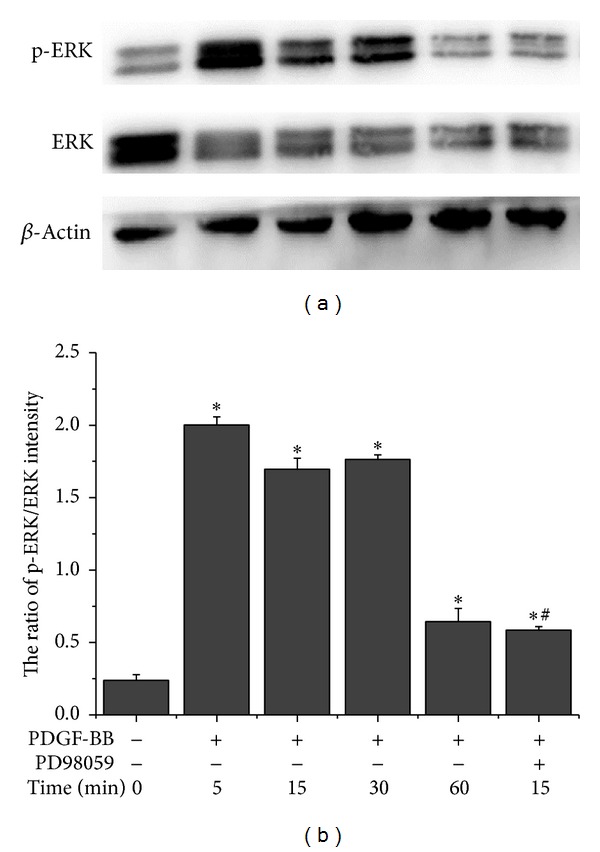
rhPDGF-BB activated ERK pathway in diabetic BMSCs. Cells were pretreated with or without 20 **μ**M PD98059 for 1 h and treated with 10 ng/mL rhPDGF-BB for the indicated periods. The unmanipulated cells were used as controls. (a) Representative image of western blotting of p-ERK, ERK, and **β**-actin (*n* = 3). (b) The ratio of p-ERK/ERK intensity were quantified by densitometry and expressed graphically. *P < 0.05, versus control, and ^#^ *P* < 0.05, versus 10 ng/mL.

To determine how rhPDGF-BB promoted the functions of diabetic BMSCs, we investigated whether ERK pathway in diabetic BMSCs was activated by rhPDGF-BB treatment. The results showed that levels of p-ERK in rhPDGF-BB treated diabetic BMSCs were unregulated significantly (Figure 3(a)). Quantitatively, the p-ERK increased approximately eightfold at 5 min, sevenfold at 15 min, and sevenfold at 30 min and decreased to twofold at 60 min in rhPDGF-BB treated group as compared with control group (Figure 3(b)). Western blot analysis indicated that 20 **μ**M PD98059 blocked ERK signaling efficiently (Figure 3(b), comparing lane 6 with lane 3).

### 3.4. rhPDGF-BB-Induced Proliferations of Diabetic BMSCs Was Inhibited by PD98059

**Figure 4:**
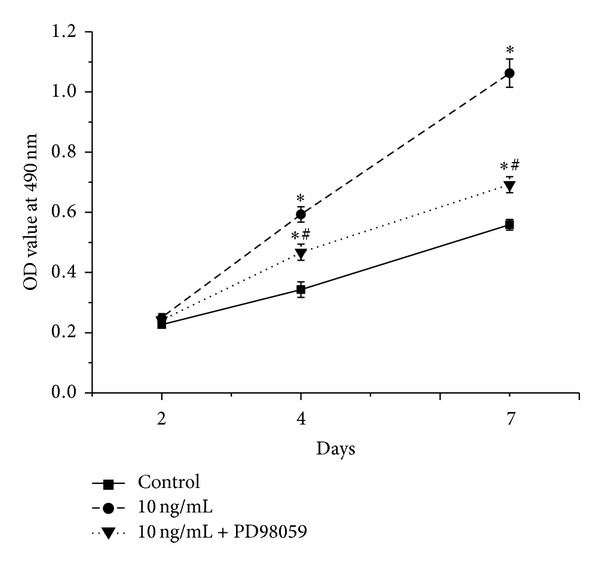
rhPDGF-BB-induced cell proliferation was inhibited by suppression of ERK pathway. Cells were pretreated with or without 20 **μ**M PD98059 for 1 h and treated with 10 ng/mL rhPDGF-BB. The unmanipulated cells were used as controls. MTT assay was assessed on days 2, 4, and 7. *P < 0.05, versus control, and ^#^ *P* < 0.05, versus 10 ng/mL.

We assessed the effect of inhibition of ERK pathway on proliferation induced by rhPDGF-BB in diabetic BMSCs. The MTT results showed that PD98059 could significantly suppress rhPDGF-BB-mediated enhancement of diabetic BMSCs proliferation at days 2, 4, and 7 (Figure 4).

### 3.5. rhPDGF-BB-Enhanced Osteogenic Differentiation of Diabetic BMSCs Was Repressed by PD98059

To elucidate whether ERK pathway was involved with the rhPDGF-BB- enhanced osteogenic differentiation of diabetic BMSCs, we performed real-time PCR, western blot analysis, ALP activity and matrix mineralization analysis.

**Figure 5:**
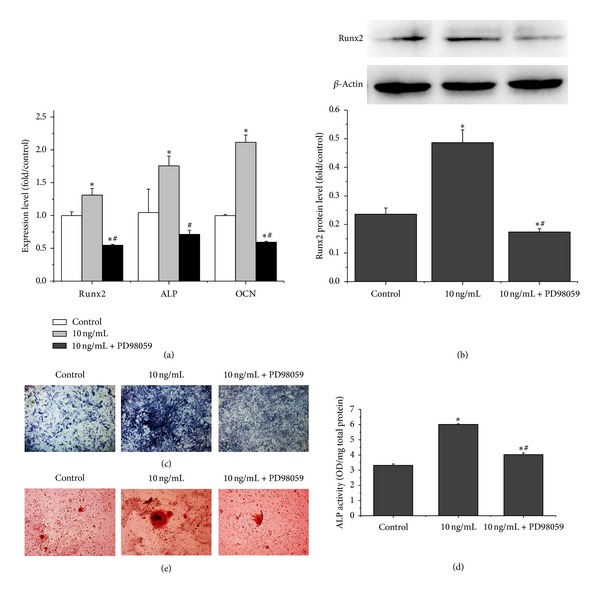
rhPDGF-BB-induced osteogenic differentiation in diabetic BMSCs was inhibited by suppression of ERK pathway. Cells were pre-treated with or without 20 **μ**M PD98059 for 1 h and treated with 10 ng/mL rhPDGF-BB. The unmanipulated cells were used as controls. (a) Real-time PCR analysis of ALP, Runx2, and OCN mRNA expression on day 7. (b) Western blot analysis for the relative Runx2 protein levels on day 7. (c) ALP staining on day 14 (16x). (d) Quantitative results of ALP assay on day 14. (e) Alizarin red staining for mineralized nodules on day 21 (50x). *P < 0.05, versus control, and ^#^ *P* < 0.05, versus 10 ng/mL.

Real-time PCR results showed that 10 ng/mL PDGF-BB group significantly increased the expression of Runx2, ALP, and OCN at day 7. Conversely, PD98059 decreased the expression of Runx2 by 45%, the ALP by 32%, and the OCN by 41% compared with 10 ng/mL rhPDGF-BB group (Figure 5(a)). In addition, western blotting demonstrated that PD98059 markedly decreased rhPDGF-BB-enhanced expression of protein Runx2 (Figure 5(b)).

ALP staining was stronger in 10 mg/mL rhPDGF-BB group than in control group, while PD98059 decreased the intensity of ALP staining compared with 10 mg/mL rhPDGF-BB group (Figure 5(c)). Meanwhile, rhPDGF-BB significantly increased ALP activity compared with control group, while PD98059 could significantly decrease rhPDGF-BB-enhanced ALP activity (Figure 5(d)). Furthermore, the Alizarin red staining showed that rhPDGF-BB significantly increased matrix mineralization compared with the control group, whereas PD98059 could significantly decrease matrix mineralization compared with 10 ng/mL group (Figure 5(e)). Collectively, these results indicated that ERK pathway is the mediator of the enhanced effect of rhPDGF-BB on osteogenesis of diabetic BMSCs.

## 4. Discussion

Diabetes is associated with a series of bone complications, which are challenging in the clinic. It is well documented that diabetes impaired osseous healing, showing as delayed union or nonunion [27]. Recently, considerable interest has focused on cell-based therapy to promote bone regeneration and repair, even in diabetes [28]. As primitive cell population, bone marrow stromal cells (BMSCs) are attractive cell sources and are crucial for bone remodeling and regeneration. However, recently, BMSCs from diabetic animals have been shown to have reduced proliferation and differentiation capacity, which may be responsible for bone disorders associated with diabetes [14, 15]. And the aforementioned problems may affect cell-based therapies for treatment of diabetic bone diseases. Therefore, modulation of diabetic BMSCs to enhance their proliferation and functions could have potential clinical implications in diabetes.

Agents such as growth factors could augment proliferation and differentiation potential of BMSCs. Impaired functions of mesenchymal stem cells by diabetes could also be enhanced by growth factors [29, 30]. Increasing evidence showed that PDGF is potent growth factor to enhance osteoblastic differentiation in many types of cell [31]. Our group previously also demonstrated that rhPDGF-BB enhanced the proliferation, differentiation, and mineralization of normal BMSCs in culture [22]. PDGF-BB has been found decreased in the fracture site in diabetic rats [17]. And study using diabetic rats has demonstrated that local administration of rhPDGF-BB could enhance the fracture healing [23]. However, so far, little is known about whether rhPDGF-BB could affect the fate of BMSCs from diabetic rats or not. In this study, the BMSCs were harvested from diabetic rats. The experimental conditions were slected to relevent to a potential autologous cell-based therapy clinical protocol. Previous studies reported that bone marrow cells derived from normal and ovariectomized rats exhibited significant differences in their osteogenic response to basic fibroblast growth factor (bFGF) and bone morphogenetic protein 2 (BMP-2) treatment [32]. So, it is necessary to study the effects of rhPDGF-BB on proliferation and osteoblastic differentiation of diabetic BMSCs.

BMSCs proliferation in response to growth factors is essential for skeletal development, bone remodeling, and fracture repair. The literature suggested that PDGF-BB is potent stimulator of cell growth [33]. Consistently, in this study, MTT assays showed that rhPDGF-BB enhanced proliferation of diabetic BMSCs in a dose- and time-dependent manner. These results were also consistent with our previous observations in normal BMSCs and demonstrated clearly that rhPDGF-BB could improve the diabetes-related proliferative impairment of BMSCs [22].

Our results also demonstrated that rhPDGF-BB significantly upregulated the mRNA levels of the osteogenic genes such as ALP, Runx2, and OCN and the protein levels of Runx2 in a dose-dependent manner. Furthermore, rhPDGF-BB increased ALP activity levels and the induction of mineralization of diabetic BMSCs in vitro. Runx2 is an early cell fate marker required for osteogenic differentiation, which could regulate downstream genes maintaining osteoblastic phenotype, such as ALP and OCN [34, 35]. ALP, a membrane bound enzyme, is a marker of early bone differentiation. OCN is a later stage protein expressed in the mature bone tissue. Altogether, our data indicates that rhPDGF-BB stimulates diabetic BMSCs differentiation and maturation. These data are in accordance with studies using cells from healthy individuals [22, 31]. These results may help to explain the enhancement of rhPDGF-BB on fracture healing in diabetic rats in a cellular and molecular basis and also suggest that rhPDGFBB could be used as a potent agent in bone disorders associated with diabetes such as poor bone defect healing and osseointegration.

We also performed studies to elucidate the mechanism of action of rhPDGF-BB on diabetic BMSCs. Our present findings showed that rhPDGF-BB directly enhanced the phosphorylation of ERK, consistent with observations in normal BMSCs [36]. It is well known that ERK pathway is crucial for regulation of cell proliferation, osteoblast differentiation, and skeletal development [37]. To demonstrate whether the induced effects of rhPDGF-BB on proliferation and osteoblast differentiation of diabetic BMSCs is dependent on ERK pathway, we used PD98059, a specific inhibitor of MEK. In this study, the results indicated that pretreatment with PD98059 blocked the rhPDGF-BB-induced ERK phosphorylation. Also, suspension of ERK by PD98059 inhibited rhPDGF-BB-stimulated enhanced proliferation in diabetic BMSCs. Moreover, PD98059 repressed rhPDGF-BB-stimulated expressions of osteogenic genes (Runx2, ALP, and OCN) and Runx2 protein, as well as ALP activity and mineralization in diabetic BMSCs. These data suggested that rhPDGF-BB regulates proliferation and osteogenic differentiation of diabetic BMSCs partially through regulation of ERK phosphorylation. It has been well documented that ERK phosphorylation enhanced osteoblast differentiation, which has been attributed to the phosphorylation of Runx2 and regulating the transcriptional activity of Runx2 [38, 39]. Runx2 is a key and early transcription factor essential for osteoblast differentiation and bone formation, which could regulate ALP and OCN. As we observed that both the protein levels and the transcriptional activity of Runx2 were upregulated by rhPDGF-BB, we consider that rhPDGF-BB may stimulate osteogenic differentiation of diabetic BMSCs partially via inducing ERK-dependent Runx2 expression, which is needed to be further confirmed. In addition, as we previously found the inhibited ERK activation was correlated with the decreased osteogenic potential observed in the diabetic BMSCs compared with normal BMSCs [14]. It is plausible to propose that rhPDGF-BB could partially reverse the detrimental effect of diabetes on BMSCs and restore their partial functional impairment.

In summary, rhPDGF-BB could enhance cell proliferation and promote differentiation and mineralization in diabetic BMSCs. Furthermore, the enhanced osteogenic functions may be partially through ERK pathway. Although future studies are needed to further clarify whether rhPDGF-BB induces the osteogenic ability of diabetic BMSCs in vivo, the present data indicate that rhPDGF-BB could be a potential therapeutic agent to partially restore proliferative and osteogenic differentiation impairments of diabetic BMSCs. And these results may suggest an effective strategy to employ the cell-based therapies in the treatment of challenging bone disorders in diabetes.
